# Web-Based Screening, Brief Intervention, and Referral to Treatment for Traumatic Stress and Alcohol Misuse Among Survivors of Sexual Assault and Intimate Partner Violence: Usability and Acceptability Study

**DOI:** 10.2196/49557

**Published:** 2024-02-15

**Authors:** Christine Hahn, Emily Tilstra-Ferrell, Selime Salim, Nada Goodrum, Alyssa Rheingold, Amanda K Gilmore, Sara Barber, Angela Moreland

**Affiliations:** 1 Department of Psychiatry and Behavioral Sciences Medical University of South Carolina Charleston, SC United States; 2 Department of Psychology University of South Carolina Charleston, SC United States; 3 Department of Health Policy & Behavioral Sciences Georgia State University Atlanta, GA United States; 4 South Carolina Coalition Against Domestic Violence and Sexual Assault Columbia, SC United States

**Keywords:** screening, brief intervention, and referral to treatment, brief intervention, intimate partner violence, sexual assault, substance use, alcohol use, mobile phone

## Abstract

**Background:**

Recent survivors of intimate partner violence (IPV) and sexual assault (SA) are at a high risk for traumatic stress and alcohol misuse. IPV and SA survivors face barriers to services for traumatic stress and alcohol misuse and have low service utilization rates. One way to increase access to services for this population is the use of web-based screening, brief intervention, and referral to treatment (SBIRT), an evidence-informed approach for early identification of traumatic stress and alcohol and drug misuse and connecting individuals to treatment.

**Objective:**

This study aims to assess the usability and acceptability of a web-based SBIRT called CHAT (Choices For Your Health After Trauma) tailored to address traumatic stress and alcohol misuse following past-year IPV, SA, or both.

**Methods:**

Phase 1 involved gathering feedback about usability and acceptability from focus groups with victim service professionals (22/52, 42%) and interviews with past-year survivors of IPV, SA, or both (13/52, 25%). Phase 2 involved gathering feedback about the acceptability of an adapted version of CHAT in an additional sample of recent survivors (17/52, 33%). Survey data on history of IPV and SA, posttraumatic stress disorder symptoms, alcohol and drug use, and service use were collected from survivors in both phases to characterize the samples. Qualitative content and thematic analyses of the interviews and focus group data were conducted using a coding template analysis comprising 6 a priori themes (usability, visual design, user engagement, content, therapeutic persuasiveness, and therapeutic alliance).

**Results:**

Six themes emerged during the focus groups and interviews related to CHAT: usability, visual design, user engagement, content, therapeutic persuasiveness, and therapeutic alliance. Phase 1 providers and survivors viewed CHAT as acceptable, easy to understand, and helpful. Participants reported that the intervention could facilitate higher engagement in this population as the web-based modality is anonymous, easily accessible, and brief. Participants offered helpful suggestions for improving CHAT by updating images, increasing content personalization, reducing text, and making users aware that the intervention is confidential. The recommendations of phase 1 participants were incorporated into CHAT. Phase 2 survivors viewed the revised intervention and found it highly acceptable (mean 4.1 out of 5, SD 1.29). A total of 4 themes encapsulated participant’s favorite aspects of CHAT: (1) content and features, (2) accessible and easy to use, (3) education, and (4) personalization. Six survivors denied disliking any aspect. The themes on recommended changes included content and features, brevity, personalization, and language access. Participants provided dissemination recommendations.

**Conclusions:**

Overall, CHAT was acceptable among victim service professionals and survivors. Positive reactions to CHAT show promise for future research investigating the efficacy and potential benefit of CHAT when integrated into services for people with traumatic stress and alcohol misuse after recent IPV and SA.

## Introduction

### Background

Sexual assault (SA) and intimate partner violence (IPV) remain major public health concerns for both women and men. In the United States, an estimated 43.6% of women and 24.8% of men experience some form of contact sexual violence in their lifetime, and 21.3% of women and 2.6% of men report being a survivor of attempted or completed rape [1]. Furthermore, more than one-third of women (36.4%) and men (33.6%) in the United States have experienced contact sexual violence, physical violence, or stalking by an intimate partner [1]. Individuals whose gender identity is transgender, gender queer, or nonbinary experience higher rates of SA and IPV compared with cisgender individuals [2,3]. Survivors of IPV and SA are at high risk for traumatic stress symptoms and alcohol misuse (ie, >4 drinks per day or 14 drinks per week for men and >3 drinks per day or 7 drinks per week for women) [4-6]. Although evidence-based treatments for traumatic stress and alcohol misuse exist, survivors of recent IPV and SA receive related services at low rates [7-9] and face many barriers to engaging in treatment [10]. Screening, brief intervention, and referral to treatment (SBIRT) is an evidence-based approach for identifying and reducing alcohol misuse, and web-based delivery shows promise in decreasing alcohol use, addressing barriers, and increasing treatment engagement among survivors of IPV and SA [11,12]. This study examined the usability of a web-based SBIRT intervention for traumatic stress and alcohol misuse tailored to recent survivors of IPV and SA.

### Traumatic Stress and Related Alcohol Misuse Among Survivors of IPV and SA

Survivors of IPV and SA are at a high risk for traumatic stress and alcohol misuse [4-6]. A nationally representative study revealed that, when accounting for sociodemographic factors such as age, ethnicity, marital status, income, and education, people who experienced IPV in the past year were more likely to have problematic use of alcohol in the past year [13]. The effect of recent IPV remained for alcohol use even when accounting for past-year mood and anxiety disorders, lifetime personality disorders, and IPV perpetration. Among a national sample of lifetime female survivors of SA, the current prevalence of alcohol use disorder (AUD) ranged from 5% to 20% depending on the type of rape (ie, forced rape, incapacitated rape, or combined type) [6], and more than half of the people who receive an SA medical forensic examination report alcohol misuse [14]. Symptoms of traumatic stress are also high after recent IPV and SA, with 57% of the lifetime survivors of IPV [15] and 74% of the past-month survivors of SA [5] reporting traumatic stress symptoms.

Self-medication or using alcohol to cope with trauma-related distress is theorized to account for high rates of alcohol misuse among survivors of IPV and SA [16-18]. Existing research supports the self-medication hypothesis among survivors of IPV and SA. The negative sequelae of exposure to IPV and SA, including posttraumatic stress disorder (PTSD), depression, and other mental health difficulties, serve as mediating factors linking IPV and SA to increased alcohol use, consistent with the self-medication hypothesis [16,17]. Qualitative data also point to alcohol use as means to cope with the emotional distress of IPV [19]. Similarly, longitudinal evidence suggests that traumatic stress symptoms predict subsequent increase in alcohol use [16].

Aligned with the self-medication hypothesis, the motivational model of alcohol use suggests that people make decisions about drinking based on the expected positive consequences (eg, avoid negative affect) and negative consequences (eg, approach positive affect) [20]. Among people with SA or IPV histories, negative affect from trauma-related distress increases the likelihood of using alcohol to cope [21,22]. Thus, increasing motivation to reduce alcohol use and teaching alternative coping strategies to use during periods of negative affect, particularly in response to trauma-related distress, recently after exposure to IPV and SA could have a substantial impact on decreasing the development and sustainment of alcohol misuse. In addition, the empowerment model, which is often applied to interventions for survivors of SA, highlights the importance of aligning a survivor’s long-term goals with their behaviors in trauma recovery [23]. Therefore, aligned with the empowerment process model, empowering survivors by giving power and control over choices about their health [24] and helping survivors to identify how their values align with their current alcohol use could enhance the likelihood of reducing use after IPV and SA.

### Mental Health Service Use for Traumatic Stress and Alcohol Misuse Among Survivors of IPV and SA

Interventions that are effective at addressing traumatic stress and co-occurring alcohol misuse are available. For example, COPE (Concurrent Treatment of PTSD and Substance Use Disorders Using Prolonged Exposure) [25] and Seeking Safety [26] are effective integrated treatments for addressing traumatic stress and alcohol misuse simultaneously. In addition, traumatic stress treatment alone (ie, Cognitive Processing Therapy) has been shown to reduce alcohol and other substance use among survivors of SA [27]. Finally, a psychoeducational video shown to survivors of recent SA during a forensic examination was found to be an effective early intervention to reduce the risk of alcohol misuse among recent survivors with a previous history of exposure to SA [28]. Although numerous treatment options for traumatic stress and alcohol misuse are available, survivors of IPV and SA face barriers to accessing treatment for these conditions. Despite high rates of alcohol misuse among survivors, it is estimated that only 20%-35% of survivors seek medical, psychiatric, or mental health services [29,30], with an even smaller subset seeking treatment for substance use disorders. People in abusive partner relationships may be hindered by their partner from seeking treatment, may not have independent financial resources, or may be subject to exacerbated violence or retribution for seeking treatment [31]. Furthermore, survivors of IPV and SA may not disclose their alcohol misuse to service providers as active substance use can be an exclusion criterion for accessing safe housing in *zero-tolerance* IPV shelters [19].

Additional barriers to treatment engagement include the lack of available mental health and substance use resources in the community, limited transportation particularly for rural residents, stigma associated with behavioral health service utilization, immigration status for undocumented survivors, and a lack of culturally sensitive services [19,31]. Stigma and discrimination associated with intersecting racial, ethnic, gender, and sexual identities can create additional obstacles to receiving care [32,33]. Furthermore, research highlights the potential for further harm when survivors seek services owing to limited training and awareness of IPV and SA-related issues among staff, which may increase fears that survivors will not be believed or will be blamed for the IPV or SA [10]. Staff in substance use disorder treatment settings report feeling ill-equipped to identify and address IPV [30]. In addition, even for survivors who access victim-related services, treatment services for alcohol misuse are typically not integrated within these settings [34], leaving a critical care gap for survivors of IPV and SA with alcohol misuse.

### Use of SBIRT for Alcohol and Drug Misuse

SBIRT is a public health–based approach to screening for alcohol misuse, assessing the level of risk, and providing an appropriate intervention that can include no intervention, brief motivational interviewing, or referral to AUD treatment [35]. SBIRT has been effectively extended to address the screening, intervention, and referral to treatment needs of survivors of trauma with alcohol misuse, known as T-SBIRT. This approach has shown promise in elevating the rates of referrals for survivors of trauma experiencing alcohol misuse to specialized mental health services [36]. SBIRT has shown efficacy in reducing alcohol use and has been applied across a range of clinical and community settings, including primary care clinics, emergency departments, outpatient medical settings, and employee assistance programs [37].

In recent years, there has been an increase in efforts to adapt SBIRT to electronic health technologies (eg, computer, web, and phone based) because it may increase disclosure of alcohol misuse owing to increased comfort and decrease provider barriers to screening and providing brief interventions [38]. However, limited research has examined web-based SBIRT as an early intervention among survivors of IPV and SA. A randomized controlled trial of a web-based SBIRT intervention for identifying and addressing IPV among women who used substances, Women Initiating New Goals of Safety, was developed and increased survivors’ likelihood of seeking follow-up care for IPV and reduced drug use at a 3-month follow-up [12]. Similarly, Brief Spousal Assault Form for the Evaluation of Risk [11], a web-based intervention for co-occurring substance use and IPV was feasible and acceptable among a sample of women presenting to the emergency department. Safe and Healthy Experiences is another computerized SBIRT intervention specific for alcohol misuse delivered on an iPad tailored for female Veterans who have lifetime experiences of SA seeking services in primary care [39]. Results from these studies support that SBIRT delivered via eHealth is feasible and acceptable; however, preliminary efficacy results on substance use outcomes were mixed.

These existing interventions are limited because they are not specific to recent IPV, SA, alcohol misuse, or traumatic stress. SBIRT may be advantageous in the months after IPV and SA because this is a period when there is a risk for patterns of alcohol misuse to intensify because of elevated trauma-related stress [5]. SBIRT was developed for use in medical settings, such as emergency care centers, clinics, and primary care, with the intention of reaching people who are at a high risk for alcohol misuse. However, most recent survivors, who would be appropriate for SBIRT given the high-risk period for alcohol misuse, do not receive related medical care [14,40,41]. Although some limited studies have identified early interventions for survivors of recent IPV and SA, there is no consensus on the best approach to early intervention following recent SA and IPV [42]. To address gaps in service provision and research, we developed a web-based SBIRT called CHAT (Choices For Your Health After Trauma), which is compatible for use on a smartphone and, therefore, has the potential to be disseminated in a variety of community settings and on social media, increasing reach. CHAT is tailored for recent survivors of IPV and SA and provides SBIRT primarily for alcohol misuse, while also screening and offering psychoeducation about traumatic stress and drug use, which commonly co-occur with alcohol misuse among survivors of SA [14,41]. The SBIRT intervention is based on the motivational model of alcohol use [20], self-medication theory [16], and the empowerment model [23] and applies principles of motivational interviewing to reduce motives to drink alcohol and increase valued living, particularly when experiencing negative affect, which are theoretical and empirically supported intervention targets for alcohol misuse. CHAT follows the core SBIRT model components. First, the intervention provides psychoeducation about alcohol use specific to IPV and SA. Next, screening and assessment of alcohol misuse, drug use, and traumatic stress symptoms are completed by administering brief validated, standardized measures (ie, Alcohol Use Disorder Identification Test–Concise, AUDIT-C [43], item 2 from the National Institute of Drug Abuse-Modified Assist [44], and the Primary Care PTSD Screen for DSM-5 [45]). Next, participants receive personalized feedback about trauma symptoms, coping with traumatic stress symptoms (eg, self-blame and nightmares), drinking quantity, recommended drinking limits, money spent on alcohol and drugs, and the impact of drinking and drugs on recovery from IPV and SA adapted from the National Institute on Alcohol Abuse and Alcoholism Rethinking Drinking [46]. In addition, brief exercises aimed at increasing motivation and healthy alternatives to alcohol use, including value identification, readiness to change ruler, goal setting, identification of coping skills, and social support adapted from Brief Spousal Assault Form for the Evaluation of Risk [11]. Finally, a personalized printable plan with all psychoeducational information and personalized goals created when using the intervention is provided with numerous referrals in the local community for traumatic stress or alcohol or drug-related services. Throughout the intervention, users are provided feedback on their responses and provided tailored recommendations for care (eg, “Your reactions are common and natural responses to violence. If these reactions are bothering you, it might be time to try therapy or other services. We will provide you with a list of providers at the end.”).

Empowerment is an essential component of recovery after IPV and SA as increased agency and control over one’s choices is associated with improved mental health outcomes [47]. Therefore, throughout the intervention, empowering imagery and themes are integrated. For example, aligned with the empowerment process model, the intervention emphasizes the importance of personal choice and autonomy and places survivors in control by providing options (eg, choices for personal goals that range from abstinence to a smaller reduction amount or choosing to not set a goal). Furthermore, by providing the intervention in a web-based format, the intervention is survivor led and self-paced, which is intended to enhance feelings of agency and choice throughout the intervention.

### This Study

IPV and SA remain highly prevalent [1-3]. Unfortunately, there is a strong bidirectional association of IPV and SA with traumatic stress and alcohol misuse [4-6]. However, many survivors of IPV and SA do not engage in the services needed for traumatic stress and alcohol misuse because of several access barriers. One avenue for addressing barriers and increasing access for this population is the use of web-based SBIRT for traumatic stress and alcohol misuse, which incorporates tailored IPV and SA content. The purpose of this study was to examine the usability and acceptability of a web-based SBIRT intervention (CHAT) designed for recent survivors of IPV and SA, adapted from previous web-based SBIRT applications [11], among a sample of victim service professionals (VSPs) within IPV and SA advocacy centers and survivors of IPV and SA. Usability testing for web-based interventions refers to formal evaluation for use within the population of interest to identify methods to receive feedback that improves the design and addresses errors in the application [48]. Testing usability of eHealth interventions using a combination of quantitative and qualitative methods is crucial for obtaining adequate feedback to improve and tailor web-based interventions for use in specific populations [48].

This study had 2 phases. The first phase involved refining CHAT by making iterative adaptations based on feedback about usability and acceptability gathered from VSPs in focus groups and people who have experienced recent IPV and SA in individual interviews. After making iterative adaptations to the intervention, the second phase was to gather feedback about the acceptability of the adapted version of CHAT in an additional sample of people who have experienced recent IPV and SA.

## Methods

### Ethical Considerations

The institutional review board (IRB) at the Medical University of South Carolina (Pro00080368) approved this study. Informed consent was obtained from all survivors before their participation in the study. A Waiver of Consent was issued by the IRB for VSPs, and all VSPs were made aware of the risk to loss of confidentiality before participating in focus groups. The provider and survivor participants in both phases were assigned a random ID to protect their anonymity. Providers were compensated with US $25 Amazon gift cards for participation in focus groups. Phase 1 survivors received US $75 Amazon gift cards as compensation. Phase 2 survivors received US $50 Amazon gift cards as compensation. Transcriptions of focus groups were deidentified and stored in a secure location.

### SBIRT Development and Design

CHAT was created on REDCap (Research Electronic Data Capture; Vanderbilt University) [49], a secure application for creating and managing surveys, that is hosted on the Medical University of South Carolina server. We chose to create CHAT on REDCap for several reasons, including low cost, accessibility among several institutions, ability to limit privileges to potential future providers and agencies interested in the intervention to help maintain the confidentiality of users, interventions that allow for personalizing intervention content such as branching and piping logic, ability to embed videos and photos into content, and available distribution methods (eg, links, URL codes, and email). We also selected REDCap because it is compatible with use on smartphones, which is a promising approach for reaching survivors of IPV and SA given the numerous barriers to accessing formal services [50].

CHAT was based on previous SBIRT interventions for survivors of traumatic events, including an SBIRT intervention for IPV and alcohol use (Brief Spousal Assault Form for the Evaluation of Risk [11] and National Institute on Alcohol Abuse and Alcoholism Rethinking Drinking [46]). The SBIRT intervention is based on the motivational model of alcohol use [20], self-medication theory [16], and the empowerment model [23] and applies principles of motivational interviewing to reduce motives to drink alcohol and increase valued living, particularly when experiencing negative affect, which are theoretical and empirically supported intervention targets for alcohol misuse. The SBIRT intervention, “CHAT” involves (1) psychoeducation about alcohol and drug use specific to IPV and SA; (2) screening and assessment for alcohol misuse, AUD, drug use, and traumatic stress symptoms using standardized measures; (3) personalized feedback about drinking quantity, recommended drinking limits, impact of drinking and drugs on recovery from SA, and money spent on alcohol and drugs; and (4) brief exercises aimed at increasing motivation and healthy alternatives to alcohol use, including value identification, readiness to change ruler, goal setting, identification of coping skills, and social support. The intervention concludes with a personalized plan and lists national and local referrals to IPV, SA, and substance use agencies. It takes approximately 20 minutes to complete. At the beginning of CHAT, users select whether they would like to view female, male, or gender nonbinary content, and branching logic provides images and drinking recommendations tailored to their preference. Piping logic is also used to personalize the intervention in several ways, including providing feedback about the quantity of the user’s alcohol use compared with recommended limits, relating the user’s reasons for drinking to potential long-term consequences (eg, “Making healthy choices about alcohol use helps me to take good care of my children”; response options range from strongly disagree to strongly agree), integrating the top values the user initially selects into motivational exercises at the end of the intervention, and providing them with a plan at the end that summarizes their selections throughout the intervention.

### Phase 1: Refinement

#### Participants

Phase 1 included 2 samples. The first sample comprised adult VSPs (22/52, 42%) who were employed at 1 of 2 local nonprofit agencies that serve people who have experienced IPV and SA and completed semistructured focus groups. The VSPs included master-level clinical providers (9/22, 41%) and victim advocates who did not have a degree in counseling (12/22, 55%); educational level of 1 participant was unknown. The average experience of working with survivors of IPV and SA was 6.95 (SD 8.4) years and ranged from 4 months to 30 years. The second sample included adult survivors of past-year SA or physical IPV who drank alcohol (13/52, 25%).

Alcohol use was selected as an inclusion criterion because it is the most common substance used among survivors of IPV and SA and is the primary focus of intervention; however, participants could also use drugs as CHAT includes content on drug use after IPV and SA. Participants who used drugs were also permitted to be included in the sample; however, this was not required. The survivors were asked to complete a web-based screener to determine study eligibility. There were no exclusion criteria other than inability or unwillingness of the participant to provide informed consent.

#### Procedure

For phase 1, a total of 3 focus groups with 7 to 8 VSPs in each group took place at the agencies. Study flyers and email invitations to participate in the study were sent to all staff at an SA advocacy program and a nonprofit for IPV. VSPs completed a paper-based survey before the interview about demographics and experiences of treating survivors as well as an additional survey completed after the interview focused on assessing perceptions of CHAT. Survey data were entered into REDCap by 2 research assistants and checked for accuracy. Overall, 69.2% (9/13) of the survivors were recruited from the community using social media advertisements and flyers to participate. Four participants were recruited from an outpatient mental health clinic that served survivors of crime. After obtaining informed consent, interviews with survivors (13/52, 25%) were conducted in person in an outpatient clinic that served survivors of crime or over a secure web-based video platform. Baseline surveys administered on REDCap were completed before conducting the interview, and a brief second survey was administered after the interview to assess perceptions of CHAT. The interviewers and focus group moderators included 1 clinical psychologist and 2 master-level service providers.

#### Quantitative Measures

##### Demographics and Background Information

Self-report surveys were used to collect demographic and background variables from survivors and VSPs. VSPs were asked to indicate the percentage of time per week they provided services related to alcohol misuse and the type of evidence-based treatment they provided (ie, “Do you provide any of the following evidence-based treatments for PTSD and/or alcohol use in your agency?”).

##### Mental Health Self-Report Measures

Phase 1 survivors were administered measures of exposure to IPV and SA, traumatic stress symptoms, alcohol and drug use, and treatment utilization.

##### SA Victimization and IPV

SA exposure among phase 1 survivors was assessed using 2 items adapted from the Trauma History Questionnaire [50]: “Has anyone ever made you have intercourse or oral or anal sex against your will?” and “Has anyone ever touched private parts of your body, or made you touch theirs, under force or threat?.” Physical IPV exposure among phase 1 and 2 survivors was assessed using an item from the HITS (Hurt, Insult, Threaten, Scream Measure; eg, Has a partner ever physically hurt you?) [51]. Survivors were asked to indicate if the IPV and SA events occurred in the lifetime or the past year.

1. *PTSD Checklist for DSM-5 (PCL-5)* [52]: The traumatic stress symptoms associated with the most distressing incident of IPV or SA among phase 1 survivors were assessed using the 20-item PCL-5. Items are rated on a 5-point scale ranging from 0 (*not at all*) to 4 (*extremely*), and a total score is created with higher scores indicating greater traumatic stress symptoms. PCL-5 scores >31 indicated clinically relevant traumatic stress symptoms [43]. Internal consistency was excellent for the PCL-5 for the phase 1 sample (Cronbach α=.92).
2. *AUDIT-C* [43]: The 3-item AUDIT-C was used to identify alcohol misuse. Survivors responded to 3-items about alcohol use (eg, How often do you have a drink containing alcohol?), with response options ranging from 0 (ie, *never/no*) to 4 (ie, *≥4 times a week/daily or almost daily*). Responses were summed, and scores of ≥3 for women and ≥4 for men were used to indicate alcohol misuse. Internal consistency was good for the AUDIT-C for the phase 1 sample (Cronbach α=.91).

#### Qualitative Measures

To assess factors related to perceptions of the intervention created to address substance misuse, a semistructured focus group discussion and qualitative interview was developed. The term interpersonal violence was defined (ie, “Interpersonal violence means physical or sexual violence such as sexual assault or domestic violence.”) for survivors and VSP participants at the start of the interview. Next, the web-based SBIRT intervention was described to survivors and VSP participants as a self-help intervention for substance use after IPV and SA.

The interview consisted of asking survivors and VSP participants up to 4 questions as they looked at each content area of the intervention, including “What are you thinking about as you look at this page?,” “What do you like about this page?,” “What don’t you like about this page?,” and “How can we make this more interesting?.” Issues related to errors in branching, options, or presentation of content were noted by the interviewer. Follow-up probes were used to clarify information provided whenever necessary. After viewing CHAT, survivors and VSP participants were asked about usefulness (eg, “Do you think this would be useful, why or why not?”; “Do you think other people with use it, why or why not?”; and “Is this something that you think people should be told about shortly after they speak to a provider about experiencing interpersonal violence, why or why not?”).

#### Data Analysis

Descriptive statistics were computed (ie, mean and SD) for participant age, PCL-5 scores, and AUDIT-C scores. Rates of past-year and lifetime exposure to IPV and SA, race, gender, and previous service use were aggregated. Clinical psychologists with expertise in qualitative methods conducted qualitative analyses.

The interviews and focus group discussions were audio-recorded and transcribed verbatim by an IRB-approved third party. Data from qualitative interviews were organized using coding template analysis [53], applying thematic and content analysis approaches using 6 themes outlined by Baumel et al [54] to examine the quality and usability of mobile apps, including usability, visual design, user engagement, content, therapeutic persuasiveness, and therapeutic alliance. Using a deductive coding strategy not only allowed for examination of themes proposed in existing usability literature but also allowed for the development of inductive categories that emerged through coding [55].

A clinical psychologist with expertise in substance use intervention development and qualitative analysis examined each line of the transcripts and mapped participant’s responses to the coding template [55,56]. More than 1 code could be applied. A second coder, who was also a clinical psychologist with expertise in interpersonal violence and substance use, reviewed responses against the coding template. Interrater discrepancies were discussed and resolved by 2 independent coders. NVivo software (version 11.1; QSR International) was used for data management and analysis. Demographics and background variables were computed using SPSS (version 27; IBM Corp [57]).

Results from formative usability trials have shown that 80% of usability issues can be identified with a sample of at least 5 people involved in usability testing [58,59]. Thus, this study was suitably powered to assess usability.

### Phase 2: Acceptability

#### Participants

The phase 2 sample included adult survivors of past-year SA, physical IPV, or both who drank alcohol (17/52, 33%). The inclusion criteria for the survivor sample of phase 2 mirrored the inclusion criteria for survivors in phase 1.

#### Procedures

For phase 2, survivors were recruited through Facebook advertisements (14/17, 82%), through Craigslist advertisements (1/17, 6%), and from an outpatient clinic that served survivors of crime (2/17, 12%). Phase 2 survivors completed the study remotely using their own devices to complete study surveys and CHAT. They completed a baseline survey on REDCap comprising questions about demographics, IPV and SA exposure, and alcohol use. Next, the survivors completed CHAT and a survey on acceptability of the intervention.

#### Measures

Demographics, descriptive characteristics, exposure to IPV and SA, alcohol misuse, and traumatic stress were gathered from phase 2 survivors using the same validated measures as in phase 1 (PCL-5, AUDIT-C, HITS, and Trauma History Questionnaire items). Internal consistency was fair for the AUDIT-C (Cronbach α=.70) and PTSD Checklist (Cronbach α=.80) in the phase 2 sample. Phase 2 survivors were also asked whether they needed care for past-year SA, IPV, alcohol use, or drug use (eg, In the past year, did you ever want or need help with any alcohol use concerns?). The following additional measures were collected from phase 2 participants:

1. *Daily Drinking Questionnaire* [60]: The Daily Drinking Questionnaire was used to assess the number of standard drinks consumed per week by phase 2 survivors (eg, “On a typical Monday, I had _ drinks.”). The mean weekly drinks were calculated.
2. *Acceptability of Intervention Measure* [61]: After viewing the web-based SBIRT intervention, phase 2 survivors completed the 4-item Acceptability of Intervention Measure. Items were rated on a 5-point scale (1=completely disagree to 5=completely agree) and averaged. Phase 2 survivors were also asked open-ended survey questions about most-liked aspects of the intervention, least-liked aspects of the intervention, and dissemination recommendations (ie, What would be the best way to inform people about the tool?).

#### Analyses

Descriptive statistics were computed in the same way as in phase 1. Participant’s brief responses to open-ended questions were coded into representative themes and subthemes. Previous research indicates that a sample size of at least 10 should suffice for acceptability testing [62]. Therefore, the phase 2 sample size had sufficient power to assess acceptability.

## Results

### Phase 1: Refinement Qualitative Results

#### Participant Demographics

VSPs’ (22/52, 42%) experience working with survivors ranged from 4 months to 30 years, and the average experience of working with survivors was 6.95 (SD 8.4) years. Two-thirds (13/22, 59%) of the participants had experience providing services to individuals engaging in alcohol misuse. Table 1 shows the demographics of the VSPs. Overall, 85% (11/13) of the phase 1 survivors reported lifetime exposure to both SA and physical IPV, and 31% (4/13) of the survivors reported both types of exposure in the past year. Phase 1 survivors reported a high prevalence of alcohol misuse, traumatic stress symptoms, and previous substance use and mental health service use (Table 1).

**Table 1:** Demographics, trauma history, and mental health information for phase 1 victim service professionals (VSPs), phase 1 survivors, and phase 2 survivors (N=52).

			Phase 1 VSPs (n=22)		Phase 1 survivors^a^ (n=13)		Phase 2 survivors^a^ (n=17)
Age (y), mean (SD)			42 (14.8)		33 (10.6)		28 (9.2)
**Gender, n (%)**							
	Women	—^b^		11 (85)		16 (94)	
	Men	—		2 (15)		1 (6)	
	Unknown	—		0		0	
**Race^c^, n (%)**							
	Black	7 (32)		2 (15)		3 (18)	
	Hispanic	1 (4)		4 (31)		2 (12)	
	White	14 (64)		11 (85)		13 (76)	
	Did not disclose	0		0		1 (6)	
**PCL-5^d^, mean (SD)**			—		47.01 (18.16)		58.12 (9.65)
	Above cutoff, n (%)	—		10 (76.9)		17 (100)	
**AUDIT-C^e^, mean (SD)**			—		6.08 (3.54)		6.88 (3.38)^f^
	Above cutoff, n (%)	—		11 (84.6)		15 (88)	
**Drug use endorsed, n (%)**			—		9 (69.2)		—
	Marijuana use endorsed	—		6 (46.3)		—	
	Previous SUD^g^ service use	—		4 (30.8)		—	
	Previous trauma-focused service use	—		8 (61.5)		—	
	Past-year SUD service use	—		—		6 (35.29)	
	Past-year mental health service use	—		—		14 (82.35)	

^a^Survivors: Survivors of intimate partner violence, sexual assault, or both.

^b^Not available.

^c^Some individuals identified as both Hispanic and White.

^d^PCL-5: Posttraumatic Stress Disorder Checklist for DSM-5.

^e^AUDIT-C: Alcohol Use Disorder Identification Test–Concise.

^f^AUDIT-C data was missing for 1 participant in phase 2.

^g^SUD: substance use disorder.

In the qualitative analysis, content from the focus groups and interviews was organized according to the evaluation categories for usability testing proposed by Baumel et al [48]. Results from survivors and providers are described within each theme (Table 2) and revised components of the intervention following these results are presented in Textbox 1.

**Table 2:** Themes and number of survivors and focus groups that discussed each theme (N=35).

Theme	Survivors (n=13), n (%)	Focus groups (out of 3 groups; n=22), n (%)
Usability	11 (85)	2 (67)
Visual design	13 (100)	3 (100)
User engagement	10 (77)	3 (100)
Content	12 (92)	3 (100)
Therapeutic persuasiveness	11 (85)	3 (100)
Therapeutic alliance	10 (77)	3 (100)

**Revised intervention components.**

**Intervention component and contents**

- Psychoeducation
  Rates of sexual assault (SA) and alcohol misuse
  Destigmatizing alcohol-involved SA
  Breathing exercise
- Assessment
  Values identification
  Alcohol use screener (Alcohol Use Disorder Identification Test–Concise)
  Past 3-month Drug Use Screener (National Institute of Drug Abuse Assist)
  Weekly money spent on alcohol and drugs
  Posttraumatic stress disorder primary care screener
  Identifying reasons for not drinking
- Personalized feedback
  Link between drinking to cope and alcohol-related problems
  Recommended drinking limits
  Comparison of money spent on alcohol and drugs with cost of common items
- Motivational interviewing
  Readiness rulers with value exercise
  Goal setting
  Coping skills identification
  Identifying how values relate to alcohol use
  Identify social support and coping strategies to use when having urges to drink
- Summary and plan
  Summary of feedback and recommended treatment referrals as indicated

#### Theme 1: Usability

Both survivors and providers rated the usability of CHAT very positively overall. VSPs and survivors reported that the intervention was easy to use and self-explanatory, stating it was “straight to the point,” “short, sweet, and user friendly,” and “clear and concise.” A survivor specifically mentioned the following:

It’s easy to navigate, easy to understand, and it’s pretty much just a self-analysis.

VSPs provided similar feedback about the usability of CHAT, with VSPs from 2 of the 3 focus groups stating positive comments. Specifically, VSPs made comments that CHAT was “very easy to understand*.*”

#### Theme 2: Visual Design

This theme describes the appearance of CHAT, including observations about the text, font size, pictures, colors, and look and feel of the activities within the application. Survivors discussed visual design slightly more than the providers, as visual design was the most discussed topic for survivors and the second most discussed topic (after content) for providers. Survivors made comments about visual design such as follows:

I like this. It kinda breaks down everything and gives you visual points to look at. Like you immediately go to what you are experiencing.

I really like the diagram because it shows what you are doing...Drinking more than the single day limit.

Another survivor described the follows:

Picture is good too. Shows people drinking, gathering like it’s fun in a way, but reading those kind of wake up calls. Like it’s not really fun.

In all VSP focus groups, the visual design of the intervention was discussed, with most comments about visual design being positive and only a few constructive recommendations about changes to visual design. Specifically, some comments that VSPs made on visual design included “I think the graphic is good*,*” “I like the colors,” and “I like this whole visual*.*” Recommendations about visual design included changing font sizes or adding pictures.

#### Theme 3: User Engagement

The theme, user engagement, describes how well the participant was able to engage with the application including how interactive and personalized the VSP and survivor participants felt that the intervention was. This included the users’ ability to engage in the activities embedded within the intervention and how often they felt that they would engage. More than half of the survivors commented on their potential engagement in the intervention, stating that they would be very willing to engage and that they would engage in the intervention often. Specifically, 1 survivor noted, “I would definitely use it. It’s easier*.*” Survivors also mentioned that they would be more likely to engage with the intervention than with people personally, as 1 survivor stated the following:

I feel like people would use it because it’d help. You may not even want to talk to your close people who are close to you. You may just want to be anonymous, and you probably would feel more comfortable with this.

Another survivor said the following:

I guess it would be the ideal thing for somebody who didn’t want to come in and talk to anybody, and just wanna do it on...just looking for information on their own, looking for help on their own.

In all the focus groups of VSPs, it was discussed that the survivors they worked with, as well as themselves, would engage with the intervention very frequently and that the intervention was easy to use. Specifically, 1 provider stated the following:

Wow! You can pull it up on any device. I would literally use this all the time

with different clients

Another provider stated the following:

It’s so great that the whole thing takes about 15 minutes. That’s nice.

In describing ways that a patient might engage, a provider stated the following:

That’s really cool that you are able to print this off and put it on your fridge.

#### Theme 4: Content

This theme described the quality and appropriateness of the content presented during the intervention. All survivors and VSPs in each focus group made positive statements about the intervention, stating that it was rooted in evidence, appropriate for themselves or their patients, and contained information that was often relevant to patients. Specifically, 1 survivor stated the following:

It think it really hits all the points. It even has God in here which is really something people don’t mention.

Another survivor stated the following:

I actually wouldn’t add anything. I love this information.

Throughout the interviews, survivors made comments about specific content that they found relevant or that they thought was important to include. In addition, VSPs discussed mostly positive comments about the content, with just a few suggestions or examples to add. For example, 1 VSP noted the strength in content by stating the following:

I love how there are so many short snippets. I thought those were all pretty powerful points.

Another VSP discussed the comprehensive nature of the content by mentioning the following:

I like that it is fairly all-encompassing. I like that there are positive reasons and then neutral reasons.

#### Theme 5: Therapeutic Persuasiveness

Therapeutic persuasiveness included comments regarding the suitability of the content provided by the intervention and whether the provider or survivor would see the content as useful in addressing substance use or trauma. All survivors described therapeutic persuasiveness and noted that the intervention would be useful in addressing these topics among survivors. Specifically, 1 survivor noted the following:

It makes you evaluate your life and see things that you maybe didn’t see while you were drinking or why you were drinking. It makes you really think about what you are doing.

Another survivor described the following:

It helps you realize how much you really are drinking. I like it because it lets you know that [values] are important and can be accomplished by changing your substance use problem. This helps you put things into perspective for how much you’re using, and if you want help it offers you options to fix those problems.

All VSP focus groups included positive comments about the therapeutic persuasiveness of the intervention. One provider described the therapeutic persuasiveness of the intervention by stating the following:

I really like the statistics. They kind of make you feel like you’re not the only one experiencing this. It’s okay that this is happening, it’s not something to be ashamed of, it’s something to deal with. It speaks to a lot of people.

#### Theme 6: Therapeutic Alliance

This theme describes whether users thought that the intervention provided support that could mirror, support, and take the place of human contact provided by an actual provider as well as whether they saw it as a resource to bolster ongoing therapy. Approximately half of the survivors discussed the therapeutic alliance gained by the intervention, with 1 survivor stating the following:

I love that the tool is helpful but also shows the places you can go to for actual, real help with your problems. Both are good.

VSPs in all 3 focus groups discussed this theme and described that the intervention would support the concepts taught in therapy and would serve as a useful addition to therapy. Specifically, 1 provider mentioned the following:

They can share the information without anybody knowing their situation. Because most times they really don’t want to share. So to be able to use this, versus verbalize it and have somebody judge them, that is much easier.

In addition, each VSP focus group discussed information on how to integrate the intervention into practice. For example, 1 VSP mentioned the following:

We get so many clients that have alcohol and drug histories. It’ll take several sessions for me to really get them to a point where they realize they are drinking/using drugs too much. But I feel like this [intervention] can get them to that point a lot quicker, where they’re more likely to at least think about [substance use] earlier on.

VSPs in each focus group also reported that this intervention would be helpful throughout treatment and potentially at different points for different clients. For example, 1 VSP described the following:

Honestly, it depends on what stage of change the client is in. Like, if they’re like “I’m not an addict or an alcoholic, I’m not going to listen to anything you say.”

Another VSP stated the following:

This [intervention] could be part of the assessment process. They come in for their mental health assessment, ask them to get there 20 minutes early to fill this out just to screen, then give them the tool to use if needed.

#### Revised Intervention Content Aligned With Results

Iterative changes to CHAT were made based on user experience during usability testing including fixing errors noted with logic branching, piping, spelling, grammar, increasing font size, and changing images based on participant suggestions. In addition, a page encouraging user to take 5 slow breaths was added based on VSPs’ suggestion that content may increase stress and adding breathing could help users engage with the intervention. Furthermore, options for gender-based content and language around recommended drinking limits and sex were adapted based on VSP recommendations. Gender-based content options were to view images of women, men, or nonbinary content. Drinking limits were explained based on biological sex because current recommended drinking limits are based on sex assigned at birth owing to physiological differences that change the way alcohol affects the body (eg, For people assigned female sex at birth, drinking more than 3 drinks on any day or 7 drinks per week is “at risk” or “heavy drinking.”).

### Phase 2: Demographics and Qualitative Results Regarding Acceptability

#### Overview

Phase 2 survivors also reported high levels of alcohol use, traumatic stress symptoms, and previous substance use and mental health services use. In phase 2, 59% (10/17) of the survivors reported both types of exposure in their lifetime, and one quarter (4/17, 23%) of the survivors endorsed past-year SA and physical IPV. Phase 2 survivors endorsed high levels of alcohol use with an average AUDIT-C score of 6.88. More than one-third (6/17, 35%) of phase 2 survivors self-reported needing care for alcohol misuse, and one-quarter (4/17, 23%) self-reported needing care for drug use. A total of 17 survivors recruited through social media and the community viewed the web-based intervention and completed surveys. On a 5-point scale (1=completely disagree to 5=completely agree), survivor participants agreed that the intervention was, on average, acceptable (mean 4.1, SD 1.29).

#### Qualitative Results

Phase 2 participants’ (17/52, 32%) favorite aspects of the intervention were encapsulated by 4 themes: content and features (6/17, 35%) such as encouraging messages, focus on reduction rather than abstinence, and ability to download a personal plan; accessibility and ease of use (6/17, 35%); psychoeducation (3/17, 18%) about alcohol, IPV, and SA; and personalization (4/17, 23%). For example, 1 survivor wrote the following:

I liked that it made a personalized plan just for me whereas in a group setting it’s geared more towards everyone.

Approximately one-third (6/17, 35%) of the survivors denied disliking any aspect of the intervention. The aspects of CHAT that could be improved were encapsulated by 3 themes: content and features (6/17, 35%), including increased focus on broader mental health, describing prevalence of IPV and SA in a more sensitive manner, increased brevity, and increased personalization; connection to immediate services (4/17, 23%); and language access (1/17, 6%). Survivors suggested 5 dissemination methods: calls, emails, and texts (2/17, 12%), podcasts (1/17, 6%), posters and flyers (1/17, 6%), social media advertisements (10/17, 58%), websites (2/17, 12%), and referrals when seeking help for IPV, SA, or substance use (3/17, 18%). For example, 1 participant offered the following suggestion for dissemination:

If anyone entering a behavioral center, drug rehabilitation center, or any mental health office showing signs of sexual trauma or substance use, the tool could be suggested to them by a medical health provider and implemented through a computer at home or cell phone. If they don’t have a cellphone or computer at home, hopefully counties can provide the computer for them to complete the tool. Social media is always a good way to promote tools to help mental health, advertisements are how I found Better Help. I have found a lot of online counseling through Google as well.

#### Revised Intervention Content Based on Phase 2 Results

**Figure 1:**
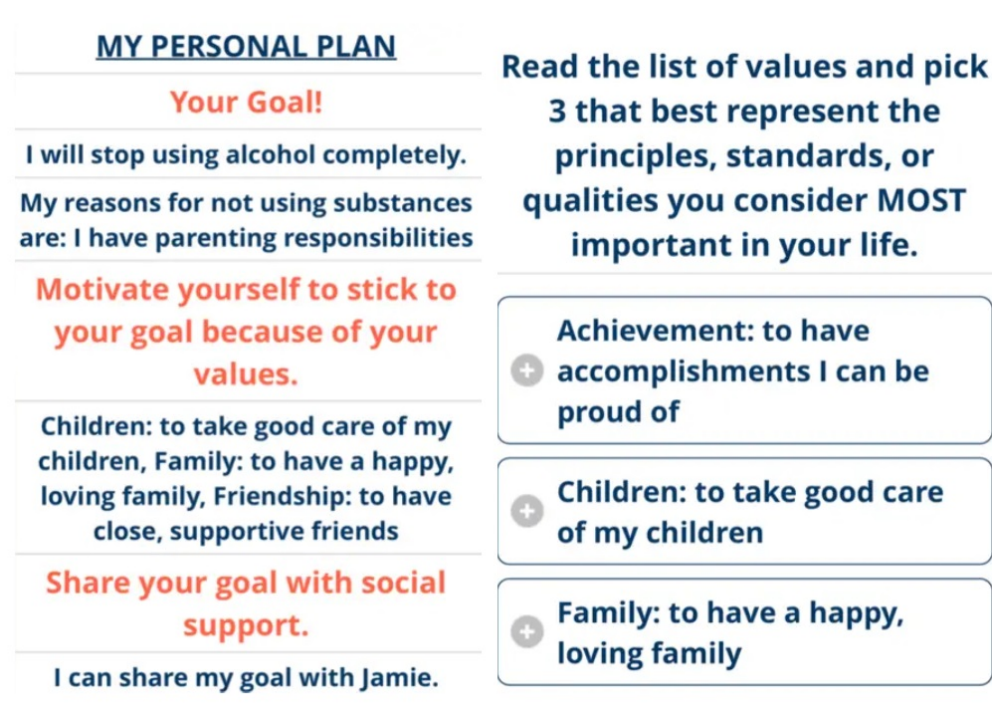
Final images of CHAT (Choices For Your Health After Trauma).

Iterative changes were made to the web-based intervention in response to participants’ suggestions (Figure 1). Images of the intervention were also updated, and images that were displayed were updated to match the preferred gender-based content selected by users at the beginning of the intervention. To improve brevity, we removed assessment of AUD symptoms and focused solely on alcohol misuse with the 3-item AUDIT-C [55]. In traditional SBIRT, the full AUDIT is used and people who report risky alcohol misuse receive brief intervention focused on reducing use and people who report more severe levels of alcohol misuse are referred to treatment. We decided to provide brief intervention and referral options based on alcohol misuse (rather than the full AUDIT) because the time after recent IPV and SA is a high-risk time for the escalation of substance use, and this minimizes the chance that we fail to provide treatment options to someone who could benefit from AUD treatment. In addition, to further personalize the intervention content to users’ drinking goals, additional content on harm reduction skills was added. Increased personalized feedback based on symptoms endorsed on the Primary Care PTSD Screen for DSM-5 was increased in the revised intervention to further interweave education about self-medication and links between alcohol misuse and traumatic stress. Finally, additional piping was used to incorporate more information about the users’ values as they relate to alcohol use. Words were also reduced for length and increased readability.

## Discussion

### Primary Findings

Survivors of IPV and SA report elevated rates of traumatic stress and alcohol misuse [4-6]. However, <35% recent survivors of IPV and SA seek services for mental health [29,30]. Higher levels of substance use are related to lower service use among survivors of recent trauma [7], which points to the importance of providing services for alcohol use *recently* after trauma exposure. This is especially important among recent survivors of IPV and SA given their uniquely high risk for alcohol misuse and drug use in this acute period [14,41]. We conducted an investigation of the usability and acceptability of a web-based SBIRT intervention for traumatic stress and alcohol misuse following an IPV and SA. Focus groups were conducted with IPV and SA service providers, and interviews were conducted with recent survivors of with the aim of gathering recommendations to improve the usability of the intervention. Following adaptation of the intervention based on this feedback, we examined the acceptability of the adapted SBIRT intervention in a second sample of recent survivors. Overall, our results suggested that service providers and recent survivors viewed the intervention as acceptable (as indicated by high ratings on the acceptability of intervention measure) and beneficial to integrate into services (as indicated by the qualitative feedback provided by VSPs and survivors). In this study, both providers and survivors commented that the intervention content was useful in addressing traumatic stress and alcohol misuse, including evaluating one’s alcohol use patterns in the context of trauma. It may be important to address the link between IPV and SA-related distress and alcohol use [16]. Although historically IPV and SA and alcohol use services have operated independently, our findings are in line with recommendations for integrated approaches that address trauma and alcohol misuse simultaneously [36,63,64].

Individuals with greater alcohol misuse may be less likely to seek care following IPV and SA [65], indicating the importance of addressing substance use–related barriers to service access. Some survivors in this study indicated that they would be more likely to engage with the intervention (rather than speak with another person) given the anonymity. This suggests that delivery of a self-directed, web-based SBIRT intervention may help circumvent common service barriers among survivors of IPV and SA related to shame, fear of the consequences of disclosure, and anticipatory stigma [10,66]. In addition, web-based delivery of SBIRT may help with barriers in clinical settings related to time constraints and staff availability [67] given the ease of access (ie, ability to use on any device), empowering content (eg, self-paced and power to decide what goals to select), and brevity (<20 minutes to complete) of the intervention. It will be important for future research to determine whether web-based SBIRT can help address some of these barriers to care if adopted into practice and to determine the most effective way of integrating this intervention into existing clinical practice.

It is imperative that the SBIRT intervention is delivered in a nonstigmatizing manner given the highly stigmatized nature of IPV, SA [68], and alcohol misuse [69]. Indeed, several survivors and providers in this study discussed the importance of ensuring that the content of the intervention is nonjudgmental and culturally sensitive. The high acceptability ratings of CHAT and previous SBIRT research [11] indicate that integrated interventions for trauma and substance misuse following recent IPV and SA can be delivered in a sensitive manner to address the important treatment needs of this population. It is crucial for future researchers developing interventions for survivors of IPV and SA to be mindful of using inclusive and nonjudgmental language. This may include explicit statements that the survivor is not to blame for the violence they have experienced, destigmatizing alcohol-involved SA by sharing statistics about the common nature of this type of assault, etc. Useful suggestions regarding design and content were made by the service providers and survivors that inform preferences for the web-based SBIRT intervention following IPV and SA focused on alcohol misuse. The intervention was modified in accordance with this feedback. The results underscored the significance of conducting usability testing with both providers and survivors. Neglecting to consider user perspectives and preferences is a key factor contributing to the low utilization of mobile mental health interventions [70,71]. Consistent with previous research indicating that privacy is an important concern for users of mobile health interventions [71], several providers and survivors in this study also discussed the need to ensure confidentiality. Therefore, interventions developed for this population may benefit from explicitly addressing issues of confidentiality. Finally, we examined the acceptability of the revised version of CHAT in a sample of recent survivors in phase 2 of this study. Research supports the effectiveness of early interventions in reducing trauma-related symptoms [72]; however, accessing health care in the weeks following assault is uncommon [73]. The current web-based SBIRT intervention was developed to address gaps in service provision after recent IPV and SA when risk for alcohol misuse and drug use is heightened owing to trauma-related distress. Survivors found the revised intervention to be acceptable and noted the personalization of the intervention as being important. Personalized care and delivery of individualized treatments is a priority for mental health care to improve the effectiveness of evidence-based interventions [74]. CHAT is an example of how a web-based SBIRT intervention can be personalized, and future research is needed to evaluate the effectiveness of this intervention in addressing needs related to substance use following IPV and SA. Taken together, our results support that, in general, recent survivors find it acceptable to be provided web-based SBIRT.

### Strengths and Limitations

The strengths of this investigation include gathering feedback from both VSPs and survivors that informed revisions of the web-based SBIRT intervention. Furthermore, the intervention included multiple substances, and the motivational content of the SBIRT intervention was personalized to refer to the substances reported by survivors (ie, alcohol use, drug use, or substance use for use of both alcohol and drugs). The high acceptability of the web-based SBIRT intervention in this study encourages examination of its efficacy in future research trials. However, the sample sizes across the 2 study phases were small, and future research with larger sample sizes is needed to test the feasibility of the web-based intervention. Inclusion criteria for survivors in this study were based on reporting of alcohol misuse. Overall, 69% (9/13) of phase 1 survivors reported drug use, with the most common drug used being marijuana (6/13, 46%). Thus, it is possible that the findings would differ if survivors were recruited based on reporting drug use. Future research should compare the acceptability, feasibility, and efficacy of the interventions among survivors reporting various types of substance use. In addition, future research may test whether allowing people who use multiple substances to select a specific substance for the motivational interventions as in the Brief Spousal Assault Form for the Evaluation of Risk intervention in the study by Choo et al [11] would improve outcomes. It is also important to note that SBIRT is most effective for people who report alcohol misuse but do not yet meet the criteria for AUD. As many recent survivors of IPV and SA may already meet the AUD or PTSD criteria before the recent trauma, the SBIRT intervention may have limited utility as an early intervention for those individuals.

It should be noted that most survivors included in this study identified as female individuals, White, and not Hispanic, and sexual orientation information was not collected. Individuals with marginalized identities based on race, ethnicity, sexual orientation, gender, and immigration status experience additional barriers to accessing services following IPV and SA [10,33,73]. In addition, experiences of identity-based stigma and discrimination may contribute to substance use [75] and exacerbate trauma-related distress following interpersonal violence [76]. Thus, it is imperative for future research to examine the usability, acceptability, and efficacy of the SBIRT intervention among survivors with diverse intersecting identities and consider experiences of stigma and marginalization. Furthermore, given the gender differences in female and male exposure to IPV and SA as well as barriers to service engagement, this study should be replicated with greater male representation in the sample. Participation was also limited to English-speaking individuals as the intervention was only available in English, and future revisions of the intervention should address language as a barrier.

All service professionals included in this study provided services within IPV and SA advocacy centers, and therefore, future evaluation of the intervention should be conducted with providers in other types of settings (eg, primary care, emergency department, and law enforcement). Furthermore, although the intervention was not designed for a single outlet for dissemination, a strength of the intervention is its flexible nature and ability to be disseminated in a wide range of settings (ie, advocacy centers, web-based forums, and emergency departments). This may increase the reach of the intervention to survivors of IPV and SA in the community who do not seek formal services. The findings in phase 2 provided crucial data to inform better ways to reach survivors and disseminate the SBIRT intervention broadly on social media and should be tested for effectiveness in reaching survivors with lower levels of service use in future research. Further implementation research is needed to understand how to integrate CHAT into specific settings (eg, rape advocacy centers). Although SBIRT is intended to be an early intervention that addresses traumatic stress symptoms and alcohol misuse and prevents the development of AUD or PTSD (because the recent months following IPV and SA are a heightened period of risk for AUD and PTSD to develop), it is possible that CHAT could also benefit nonrecent survivors. Future research should expand to apply the SBIRT model to nonrecent survivors and examine whether a broader application is beneficial. In addition, the web-based intervention had limitations in terms of referral and treatment owing to its brevity. Moreover, it lacked a follow-up mechanism to assist individuals in overcoming barriers to accessing referrals. This is a weakness of the current SBIRT intervention. Future research should focus on increasing the robustness of the referral to treatment component. Adding a support person to follow-up with intervention users might be an important component for future development [77]. For example, 1 survivor participant recommended, “Definitely follow ups, like a call, would help.”

In conclusion, results from this study among VSPs and survivors support the usability and acceptability of a web-based SBIRT intervention designed for traumatic stress and alcohol misuse among recent survivors of IPV and SA. Future research should include samples with greater diversity and address the barriers related to English language proficiency. Overall, the findings encourage future examinations of the efficacy of web-based SBIRT for co-occurring traumatic stress and alcohol misuse among recent survivors.

## Abbreviations

AUD: alcohol use disorder
AUDIT-C: Alcohol Use Disorder Identification Test–Concise
CHAT: Choices For Your Health After Trauma
COPE: Concurrent Treatment of PTSD and Substance Use Disorders Using Prolonged Exposure
HITS: Hurt, Insult, Threaten, Scream Measure
IPV: intimate partner violence
IRB: institutional review board
PCL-5: Posttraumatic Stress Disorder Checklist for DSM-5
PTSD: posttraumatic stress disorder
REDCap: Research Electronic Data Capture
SA: sexual assault
SBIRT: screening, brief intervention, and referral to treatment
VSP: victim service professional

## Data Availability

Deidentified quantitative data sets generated or analyzed during this study are available from the corresponding author upon reasonable request.
